# Supplementary material for: Are carbon emissions trading and green financial instruments synergistic? -Comprehensive quantitative research based on content analysis
Source: PLoS One. 2024 Mar 7;19(3):e0298601. doi: 10.1371/journal.pone.0298601 (PMC10919601; doi:10.1371/journal.pone.0298601)
Supplement: S1 Data — (ZIP) [file pone.0298601.s001.zip › Data/地级市Z_F工作报告.zip!/╔╜╬≈/│ñ╓╬╩╨/└┤╫╘╣1⁄2╓┌║┼í╛┬φ┐╦╩2╛▌═°í┐_2010.docx]

各位代表：

现在,我代表市人民政府向大会作报告,请予审议,并请市政协各位委员和其他列席人员提出意见。

一、2009年工作回顾

2009年是我市在挑战中克难奋进、考验中化危为机的一年。面对国际金融危机的严重冲击，在市委的坚强领导下，在市人大、市政协的监督支持下，深入贯彻落实科学发展观，坚决执行中央和省“三保”、“三个发展”的政策措施，大力实施“四位一体”发展战略，坚定信心，迎难而上，顽强拼搏，全市呈现出经济较快发展、民生持续改善、社会和谐稳定的良好局面。地区生产总值完成775.3亿元，同比增长10%；财政总收入177.9亿元，增长11.5%；一般预算收入69.6亿元，增长11.1%；城镇居民人均可支配收入15494元，增长8.5%；农民人均纯收入5337元，增长8%。市十二届人大四次会议提出的各项目标任务圆满完成，市政府向全市人民承诺的10件实事全部兑现！

一年来，我们重点做了九个方面工作：

（一）千方百计保持经济平稳较快增长。把项目建设和帮扶企业作为保增长的重点，确定了以“双十工程”为龙头的重点项目826项，总投资2000亿元，名列全省第二；成立项目建设指挥部和服务组，设立政府推进项目一线工作室，为项目建设提供零距离服务，全年竣工项目550个，是我市竣工项目最多的一年。制定保增长、促转型“双十条”措施，认真落实“五缓四减三补贴”政策；成立企业帮扶服务中心，开通服务企业热线电话；每月对经济运行和安全生产实行“双监测、双例会、双通报”；重点抓好“三百企业”发展；成立全省首家企业信用担保联盟，组建小额贷款公司，全年新增贷款77.5亿元，增长19.5%。

（二）排除万难推进煤炭资源整合和煤矿企业兼并重组。按照集团规模化、经营产业化、利益责任化、安全本质化的原则和政府主导、企业主体、市场运作、依法推动的办法，义无反顾推进煤炭资源整合和煤矿企业兼并重组。整合后全市共有22个主体、110座煤矿，煤矿数量减少63%，资源量增长68.8%，产能增长23.7%，单井平均规模达到95万吨，提高2.3倍，全部实行机械化开采。整合重组工作取得重大突破性成果，得到了省委、省政府、中央媒体采访团和受国务院委托的国家发改委调研组的表扬与肯定。

（三）抢抓机遇调结构促转型。加大对传统产业的技术改造力度，技改投资增长58.3%。大力发展战略性新兴产业，开工建设了全球第一条垂直整合的LED全产业链；LED封装项目当年开工建设、当年投产见效，达利食品一期工程竣工投产；10万辆微车生产线、康宝血液制品扩产等一批新兴产业项目进展顺利。技术改造和新兴产业对经济的贡献率提高3个百分点。大力推进现代服务业，开工建设了长治物流中心，商务会展、中介服务等生产性服务业快速发展。社会消费品零售总额244.8亿元，增长21.7%，农村消费增幅快于城市1.2个百分点。成功举办第三届长治国际攀岩节，全市旅游总收入增长23.8%，荣获中国优秀旅游城市称号。

（四）下大气力强化“三农”工作。全面落实各项强农惠农政策，各级财政对“三农”的投入增长15.5%。积极推进农业产业化，新上农业产业化项目65个，建成国家级农业标准化示范区37个，培育优势农产品生产基地10个。农民专业合作社达到3342个，居全省第一。农业综合生产能力进一步提高，粮食总产量在大灾之年完成11.7亿公斤，达到中等年景水平。农村“五个全覆盖工程”进展顺利，新农村建设连续两年受到省委、省政府表彰。开展返乡农民工就业创业“春风行动”，10.4万农民工实现就业。制定出台《长治市“1+5”城市群建设行动纲要》，实施“151”推进策略，开工建设“1+5”环城高速公路和天然气管道输送工程，城乡一体化发展取得新成效。

（五）创新举措加强安全生产。扎实推进本质安全型城市创建，建立评价指标体系，出台责任追究办法，在39个单位开展试点。认真落实安全生产各项制度措施，不断强化煤矿安全生产“两改三监督”，搞好煤矿整合重组期间的安全生产，制定“五五包保”办法，煤矿“七长”全部到位履行职责。全市高危企业全部建立安全生产指挥中心，形成以安全为中心的企业运行机制。切实搞好安全生产和社会稳定“双排查”，在各领域、各行业全面开展安全生产专项整治，全市安全生产形势持续稳定好转。亿元地区生产总值死亡率下降39%；地方煤矿百万吨死亡率下降27%；工矿企业连续七年没有发生死亡6人以上的重大事故，被省政府表彰为全省惟一的安全生产模范市。

（六）持之以恒改善生态环境。深入开展蓝天碧水工程，强化上党盆地和长治湿地保护，启动浊漳河流域综合治理工程。加大节能减排工作力度，坚决淘汰落后产能，节能工作连续三年名列全省第一。建成一批城镇集中供热、污水和垃圾处理等基础设施，提前一年实现县县都有污水处理厂的目标，二氧化硫、化学需氧量排放量降幅均完成省政府下达的目标任务。去年市区空气质量在全国113个重点监控城市中的排名由23位前移到21位，名列华北地区第一。在省政府城市环境综合整治定量考核中连续八年名列全省第一。全市投入造林绿化资金11.8亿元，植树7855万株，人均24株，成活率达到95%以上。城市绿化覆盖率达到47.8%,提高1个百分点。成功举办了全国造林绿化现场会，荣获全省林业建设突出贡献奖。

（七）有力有序推进改革开放。长钢与首钢成功重组，实现了钢铁行业的历史性突破。长治轴承厂等市属25户国有企业改革加快推进。市县政府机构改革基本完成，事业单位分类改革进展顺利，文化广电体制改革全面完成，集体林权制度改革试点工作圆满完成。对外开放不断扩大，参加了中博会、农博会等大型招商活动，实际引进各类外来资金95.5亿元。科技创新能力持续增强，第六次荣获全国科技进步先进城市称号，被国家科技部等十九部委确定为全省惟一的国家可持续发展实验区。

（八）加大力度建设和谐长治。大力实施“五大惠民工程”，继续实行公办职业高中和普通高中家庭困难学生免学费政策。城镇新增就业3.9万人，城镇登记失业率2.16%，低于全国全省控制水平。县乡村三级卫生机构达标率88.7%，提高12.7个百分点；新农合农民参合率92.6%，提高2.6个百分点。17.9万城乡低保对象实现应保尽保，在全省率先具备了农村五保户100%集中供养条件，在长治和屯留两县启动了新型农村养老保险试点。解决了1050户农村困难群众的住房问题，开工建设经济适用房、廉租房和棚户区改造住房76.5万平方米、10049套。认真落实人口和计划生育各项奖励扶持政策，人口自然增长率为4.94‰。新闻出版、广播影视、文艺表演进一步繁荣。全市财政对教育、卫生、科技、文化的投入增长19.4%。全民健身运动广泛开展。双拥共建、国防教育、民兵预备役、人民防空工作进一步巩固。老龄、妇女儿童、残疾人事业健康发展。文物保护、外事侨务、防震减灾、气象、统计、档案、保密、地方志等各项工作取得新成绩。

（九）扎实推进民主法制和精神文明建设。认真执行市人大及其常委会的各项决议，积极支持人民政协工作，高度重视人大代表建议和政协委员提案办理工作，办复率达到100%。主动加强与各民主党派、工商联、无党派人士和人民团体的联系。全面贯彻党的民族宗教政策，被国务院表彰为全国民族团结进步模范集体。坚持依法行政，进一步扩大政府信息公开范围，强化行政监察和审计监督，党风廉政建设和反腐败工作取得新成效。加强综合治理和信访工作，认真办理书记市长公开电话反映的问题，严厉打击各类严重刑事犯罪活动，妥善处置各类突发事件，有效应对强降雪灾害,全力防范甲型H1N1流感疫情,积极做好国庆60周年安保工作。扎实开展文明城市创建活动，再次荣获创建全国文明城市先进市称号。

各位代表，过去一年我们所取得的成绩，是省委、省政府和市委正确领导的结果，是全市上下弘扬认真、团结、务实作风的结果。在此，我代表市人民政府，向广大工人、农民、知识分子和各级干部，向各民主党派、工商联、无党派人士和人民团体，向驻市部队广大指战员、武警官兵、公安干警，向所有关心支持长治发展的各界朋友，致以崇高的敬意和衷心的感谢！

在充分肯定成绩的同时，我们清醒地看到，国际金融危机不仅对我市经济增长带来了影响，更使经济运行中长期积累的一些结构性、体制性矛盾凸显：新兴产业和现代服务业比重偏低，自主创新能力不强；转型发展、节能减排的任务极为繁重；统筹城乡一体化发展亟待突破，农民持续增收困难较多；劳动就业、社会保障、收入分配、住房、教育、医疗等方面还存在不少问题；部分政府工作人员责任心不强，一些重点工作推进不够理想，个别领域腐败现象仍然存在，政府管理创新还需加大力度。对这些问题，我们将采取更加有力的措施，认真加以解决。

二、2010年主要任务

2010年是完成“十一五”规划、谋划“十二五”发展的关键之年。综合判断，今年经济发展形势总体将好于去年，但许多不确定因素依然存在。我们必须深刻认识国内外形势的复杂性，增强忧患意识和风险意识，更加积极地抢抓机遇，更加科学地谋划发展，更加有效地破解难题。

今年政府工作的指导思想是：坚持以邓小平理论和“三个代表”重要思想为指导，深入贯彻科学发展观，认真落实党的十七大和十七届三中、四中全会精神，按照省委“三个发展”的部署，紧紧围绕实施“四位一体”发展战略，突出保发展、促转型、惠民生的基本要求，以转变发展方式为主线，推进结构调整、推进城乡一体化、推进生态文明建设、推进社会建设、推进改革创新、推进城市品牌创建，进一步加快在全省率先发展的步伐。

今年经济社会发展主要目标是：地区生产总值增长10%；全社会固定资产投资增长25%；社会消费品零售总额增长16%；财政总收入和一般预算收入均增长8%；城镇居民人均可支配收入和农民人均纯收入均增长9%；居民消费价格涨幅控制在3%以内；万元地区生产总值综合能耗下降3.5%；二氧化硫、化学需氧量排放量分别下降1%和1.5%；城镇登记失业率控制在4.2%以内；人口自然增长率控制在5‰以内；亿元地区生产总值死亡率控制在0.23以下。

今年要重点抓好九个方面工作：

（一）努力保持经济平稳较快发展。巩固经济回升向好势头，保持经济平稳较快发展是一项重要任务。我们必须认真贯彻落实中央和省各项政策措施，进一步增强投资、消费和进出口对经济增长的拉动作用。

继续增加投资。今年全市确定项目670个，总投资2284亿元，当年计划完成投资530亿元，重点投向重大基础设施和“三农”、改善民生、节能环保、自主创新、技术改造等领域。在全力抓好“双十工程”的同时，倾力建设一批打基础、促转型、增后劲、利长远的产业项目。

继续扩大消费。进一步提高最低工资标准。积极实施和完善鼓励消费的各项政策措施，落实家电、汽车以旧换新和汽车、摩托车下乡政策。积极培育信息、文化、健身、培训、养老、家政服务等消费热点，促进消费结构优化升级。努力扩大出口规模，大力实施出口市场多元化战略。整顿和规范市场秩序，加强价格监管，实行严格的产品质量管理制度，努力营造诚实守信的消费环境。

继续加大项目推进和扶持企业力度。对新开工的116个重点项目实施“四制、三审、一复核”的管理制度。充分发挥政府推进项目一线工作室作用，及时办理立项、土地、环评等方面的审批手续，确保重点项目顺利推进。继续落实保增长、促转型“双十条”措施，坚持对经济运行和安全生产实行“双监测、双例会、双通报”，进一步发挥企业帮扶服务中心作用，建立服务企业网络“直通车”。鼓励金融创新，积极发挥企业信用担保联盟和小额贷款公司的作用，缓解中小企业融资难。

（二）加快推进经济发展方式转变。转变经济发展方式刻不容缓。我们必须大力推进产业结构的战略性调整，加快发展低碳经济，促进高碳产业低碳化，推动经济发展进入创新驱动、内生增长的轨道，努力在经济发展方式转变上走在全省前列。

加快与国家和省产业政策的对接。按照市委九届十次全会精神，制定出台《长治市可持续发展实验区建设推进方案》，力争成为全省资源型地区可持续发展的先导区，在发展规划、环境等方面争取更大支持。加速推进调整经济结构的“十大转变”和“1+8”产业振兴行动。进一步研究制定民间资本项目核准、融资服务、财税扶持等具体办法，拓宽民间资本投资实体经济的渠道，引导民间资本与高端人才和先进技术的结合，充分发挥民间资本在转型发展中的作用。

加快战略性新兴产业的发展。加速建设300万吨煤基合成油、15万吨HF节能环保燃油乳化、8000吨电子级和光伏级多晶硅等项目，尽快投产达效，成为新的增长点；加速建设LED二期工程，积极打造省级光电园区；加速建设10万辆微型汽车、高效节能电机等项目,做强先进装备制造业；加速建设康宝血制品深度开发和新型疫苗、振东复方苦参注射液和中药饮片加工等项目，发展生物医药业和现代中药业；加速建设金泽玉米深加工、达利食品二期工程、沁州黄小米、瑞强养殖、上党保健食品等项目，壮大绿色食品工业。

加快传统产业的改造升级。全面推进循环经济发展，延伸煤炭、焦炭、电力、冶金、化工、建材、机械等产业产品链条。推进北京大学·山西新能源新工业产业化中心首批落地项目建设。善始善终完成好煤炭资源整合和煤矿企业兼并重组的各项工作，重点推进71座煤矿扩能改造。搞好潞宝7.63米大型焦炉、天脊60万吨甲醇、首钢长钢公司扩能改造等项目建设，提升产业素质，增强企业发展内生力。

加快现代服务业的发展。做大做强我市具有潜在优势的生产性服务业、文化产业和旅游业。启动物联网建设，发展电子商务、会展商务，加快改造升级以城区十字街为轴心的商贸集聚区。提高金融业服务水平，引进浦发、华夏等股份制金融机构，发展村镇银行、小额贷款公司、担保公司，加强银企对接、银农对接。着力提升信息服务业，推进电信网、广播电视网和互联网融合发展。发展营销策划、品牌和包装设计等产业，培育影视、动漫、演艺，特别是地方戏曲等文化产业。深度开发各类旅游资源，精心打造八路军红色之旅、北方水城休闲度假、上党古文化、太行山大峡谷、太行水乡等旅游品牌，办好亚洲攀岩锦标赛。

加快科技创新和节能减排。突出企业主体地位，推动产学研融合，促进科技、标准、品牌与产业同步发展。打好节能减排攻坚战，坚决淘汰落后产能，严格控制新增能耗，做好全市100户重点企业节能减排工程建设和监测监管工作。规划建构区域循环经济系统，建立“资源—废料—原料”综合利用的生态工业园区。深入推进蓝天碧水工程，确保建成区环境空气质量稳定达到二级标准，确保各县(市、区)污水处理率达到95%以上，确保“十一五”节能减排任务圆满完成。

（三）继续强化社会主义新农村建设。现代化的关键是农村现代化。我们必须真情实意地关心农民、支持农业，做好农村各项工作。

进一步提高农业产业化水平。以增加农民收入为中心，依托全国农业综合标准化示范市的优势，拓展“龙头企业+基地+合作社+农户+金融”的现代农业发展方式，抓好100个农产品加工龙头企业，发展10个有机食品生产基地，扶持300个农民专业合作示范社。大力发展设施农业，有效提高农业效益和竞争力。启动实施农产品加工龙头企业“1231”工程。

进一步强化环农服务体系。继续推进万村千乡市场网、新农村现代流通服务网、放心粮油网“三网合一”，新建300家连锁农家店，建设改造14家放心粮油配送中心、220家示范店、1000家经销网点。搞好20个特色农产品产地批发市场和150个乡镇农产品综合批发市场建设。推进农业科技入户工程，建立农村流动农技站制度，公开招聘一批农村技师，开展农业科技帮扶工作。建设市级和6个县级农产品质量安全检验检测中心。以农业科技示范园区为载体，大力推广生态种养、秸秆利用、户用沼气等循环农业模式，形成种养加、储运销一条龙的产业链条。

进一步增强农业综合生产能力。实行最严格的耕地保护制度，确保耕地占补平衡。继续抓好农田水利基本建设，建设改造一批大中型灌区，完成屯绛、鲍家河等5个水库的除险加固，力争吴家庄水库开工建设，加快屯谷水库、永和水电站、氵朋头水电站3个新水源工程的前期工作。大力发展旱作节水农业，提高农机装备和农业机械化水平。

进一步加大强农惠农力度。全面完成省政府确定的农村“五个全覆盖”，解决30万农村人口的安全饮水问题，新增21个建制村的通村水泥（油）路139.8公里。编制全市新农村建设总体规划，抓好239个省级重点推进村、300个市级整治村建设。加大扶贫开发力度，完成133个贫困村整村推进和5000人的移民搬迁任务。不断加大对农业的投入，全面落实粮食、良种、农资、农机具等各种补贴以及我市出台的退耕还林还湿补贴、发展有机食品奖补等政策措施。实施“阳光培训”工程，发挥好农民工服务中心的作用，加大外出务工农民的就业指导、服务和维权力度，使农民在家能致富，出门能挣钱，权益有保障。

（四）加速城镇化发展进程。城镇化是扩大内需、转变发展方式的战略重点，是解决“三农”问题的重要途径，我们必须高度重视城乡统筹发展。

全面落实《“1+5”城市群建设行动纲要》。以“一中心四纽带”为重点，加快市区公共资源向周边县城延伸步伐，构筑环绕市区的路网、电网、水网、气网、通信网和绿化网等基础设施网络，增强城市综合承载能力。探索建立“1+5”城市群的综合调控机制。加快“1+5”城市群5个县市的撤县设区申报工作，争取国务院批准我市为较大城市。

全面实施“151”推进策略。统筹推进主城区、县城和小城镇发展，加快构筑“双城、双镇、双村”格局。推进户籍制度改革，放宽城镇落户条件，鼓励引导有条件的农民到城镇务工创业，将全市城镇化率提高1至2个百分点。

全面提升城市建设管理水平。加快完善新区起步区基础设施。开工建设紫金西街立交桥等一批城建工程，搞好北外环、南外环等8条城市道路照明工程。加快建设国电长治热电厂热电联供、长北污水处理厂、潞安能化总部、会展中心、商务大厦等工程。确保新闻中心、体育中心、客运东站等工程竣工。改善老旧小区、街巷胡同、城中村、城乡结合部的居住环境。推进“数字长治”建设，完善市对县（市、区）的管理方式，发挥街道、乡镇的基础管理作用，提高城市管理水平。巩固国家卫生城市创建成果，积极做好迎检工作，力争再有4个县进入国家卫生县城行列。

全面打造绿色长治城市品牌。以创建全国绿化模范城市、国家森林城市、国家生态园林城市、国家环保模范城市、宜居城市为载体，大力实施生态兴市战略。城市绿化重点抓好老顶山火烧植被恢复、漳泽湖环湖林带建设、重要节点绿地提档和垂直绿化；乡村绿化重点抓好环村绿化、房前屋后四旁绿化、庭院绿化；通道绿化重点抓好“1+5”城市群景观林带建设、主要通道两旁采石边坡植被恢复。今年全市森林覆盖率提高2个百分点。深入推进生态环境综合整治，制定出台浊漳河、沁河流域水环境综合整治规划，推进浊漳河流域生态环境治理和修复工程。抓好城市环城水系综合治理，加强上党盆地和长治湿地保护，全面恢复“两地”生态功能，努力为全市人民创造“天蓝地绿人和气”的宜居环境。

（五）更加重视保障和改善民生。保障和改善民生是执政之本，是又好又快发展的动力之源，是促进社会和谐的稳定之基。我们必须把保障和改善民生作为一切工作的出发点和落脚点。

不断提高社会保障水平。探索建立城镇居民养老保险制度，提高企业退休人员基本养老金，做好养老保险关系转移接续工作。扩大新型农村社会养老保险试点，覆盖农民达到40.1万人。提高城镇居民基本医疗保险财政补助标准和筹资水平，加快解决关闭破产企业退休人员和困难企业职工的医疗保障问题。进一步提高城乡低保水平和优抚对象抚恤、生活补助标准。大力发展慈善事业。

不断扩大和促进就业。构建统一的人力资源市场和公共就业服务平台，完善引导和扶持政策，千方百计拓宽就业渠道。切实做好高校毕业生就业工作，建立就业补贴机制，激励自主创业带动就业。继续开展就业援助，稳步扩大就业总量。全面推进劳动合同制度，维护劳动关系和谐稳定。在长治职业技术学院、市高级技工学校等高职（大专）院校增设LED、微车制造、HF燃油乳化等新型实用专业，为我市战略性新兴产业发展培养成千上万的技术人才。

不断推进教育公平。健全义务教育投入保障机制，保质、保量、按时完成198.7万平方米的中小学校舍改造加固任务。加强学前教育、特殊教育和成人教育。抓好城镇教师支援农村教育工作，引导城市优质中小学校到基层和边远山区联合办学。继续落实中等职业学校和普通高中免学费政策，为2万名职业高中生、3000名涉农专业和家庭贫困的中专生免除学费，普通高中在校生免学费比例达到20%以上。整合职业教育资源，加快长治教育园区建设。

不断发展医疗卫生事业。整合公共卫生服务资源，落实基本药物制度，启动市人民医院、长子县人民医院改革试点工作，提高公共卫生管理和服务水平。改造市人民医院、市二院等门诊和住院大楼，完成8所县级医院、4所乡镇卫生院和5个社区卫生服务中心建设，推进基层卫生所（站）“双千”工程。落实国家9项基本公共卫生和6项重大公共卫生服务项目，增强应对突发公共卫生事件的能力。提高新农合保障水平，人均筹资标准提高到150元，参合率达到95%以上，全面推行门诊统筹。抓好计生工作，实施“好娃娃”工程。

不断改善城乡群众居住条件。采取集中新建、分散配建、政府收购等多种渠道增加保障性住房。开工建设保障性住房23.24万平方米、3663套，完成城市、工矿棚户区改造住房41.5万平方米、4481套。坚决落实中央和省各项调控政策，强化房地产市场监管，抑制房价过快上涨，打击圈地不建、捂盘惜售、哄抬房价等违法违规行为，促进房地产市场健康发展。

（六）稳定提高安全发展水平。安全稳定是做好一切工作的底线。我们必须以不动摇、不放松、不懈怠的态度，全力抓好安全生产，实现安全生产形势由明显好转向稳定好转的转变。

深入创建本质安全型城市。深化安全生产领导体制、投入体制、管理体制三项改革。扩大创建本质安全型城市试点范围，进一步落实试点行动纲要和评价指标体系。扎实开展平安乡村创建活动，加快省级应急救援基地建设，提高安全保障能力。严格推行高危行业用工安全培训制度，提高职工的安全素质，使本质安全型城市创建活动取得新进展。

深入开展安全专项整治。重点抓好非煤矿山、危化物品、民爆物品、建筑施工、森林防火、消防隐患、特种设备、食品药品等领域的安全专项整治。抓好道路交通安全，加快制定城市道路交通专项规划，创新网格化、勤务化的管理机制，严厉打击交通违法违规行为。突出抓好隐患排查，对重大隐患实行分级管理、挂牌督办，盯紧看死，直到隐患彻底整改；突出抓好一线落实，把监督检查和安全执法关口前移、重心下移，切实加强生产一线的现场监管；突出抓好煤矿瓦斯、煤矿防治水安全整治。

深入创新安全监管举措。适应煤矿整合重组后现代化矿井的发展形势，建立政府监管、煤矿负责、兼并主体承诺担保的安全管理新秩序。严格实行法人安全生产承诺制度和安全生产事故零报告制度，扩大煤矿企业“5173”安全工程范围，进一步推进安全指挥中心的规范化运作，强化安全责任新落实。不折不扣落实“五五包保”、煤矿用工全员培训等制度，重点加强在建矿井和技改矿井的安全监管，实行安全监管新办法。强化安全隐患事前责任追究，进一步严格安全问责。

（七）积极推进改革创新。面对工业化、信息化、城镇化、市场化、国际化深入发展的新形势，我们必须以更加积极主动、更加切实有效的措施，加大改革开放力度，不断创新体制机制。

深化行政管理体制改革。继续推进行政审批制度改革，健全政府部门绩效评估指标体系。改革财税管理体制，全面实行乡财县管乡用财政管理体制。优化财税结构，切实增强财政增收的稳定性和可持续性。强化政府性债务管理，控制债务规模，防范债务风险。积极稳妥地推进事业单位分类改革，做好事业单位绩效工资改革各项工作。

深化企业改革。加快市潞苑建材、陶瓷厂等国有企业改制步伐。推动中小企业特别是创新、创业型企业加快发展，加大财税扶持力度，改进金融服务体系，切实减轻中小企业负担。改善非公有制经济发展环境，破除各种壁垒，创新服务方式，切实解决制约民营经济发展的市场准入、资金融通、自主创新、人才引进、项目审批等突出问题。

深化农村综合改革。创新土地流转方式和途径，规范土地流转合同管理，推进土地适度规模经营。扩大集体林权制度改革范围，完成60%以上集体林地的主体改革任务。引导农业龙头企业与农民建立稳定的利益联结关系，支持农民专业合作社和产业带头人队伍成长壮大。

深化对外开放。创新招商选资和技术引进机制，鼓励外商外资参与产业结构调整、基础设施建设和重大民生工程，吸引市外资金投向高端制造、高新技术、新能源、节能环保和现代服务业。创新人才强市办法，不拘一格选人才、引人才、奖人才，鼓励高端人才来我市干事创业。创新对外经济技术合作交流途径，支持有条件的企业对外投资，支持对外工程承包和劳务输出，巩固发展与国内外友好城市的合作交流，加强与中原经济协作区、环渤海地区的交流合作，落实好与上海松江区的产业转移承接工作。改革中博会、能博会、农博会等会展活动的参展工作。推动企业上市，加大长治海关筹建力度。

（八）推进民主法制和精神文明建设。民主法制和精神文明建设是做好全部工作的支撑和保障，我们必须一以贯之、坚持不懈地抓紧抓好。

加强依法治市。严格依照法定权限和程序履行职责，自觉接受人大、政协和社会各界的监督，认真听取各民主党派、工商联、无党派人士的意见和建议。加强基层民主建设，健全政务公开、企务公开、村务公开和农村“四议两公开”等制度，充分保障人民群众的知情权、参与权、表达权、监督权。支持工会、共青团、妇联和科协等群团组织的工作。支持法院、检察院依法独立行使审判权、检察权。加强政府法制工作，全面落实“五五”普法规划，研究制定“六五”普法规划，增强公职人员的依法办事能力和公民的法律素质。

加强社会管理。健全维护群众权益民意诉求机制，进一步加强信访工作，充分发挥书记市长公开电话的作用。健全社会治安防控综合治理机制，不断完善社会治安防控体系和公共安全保障体系，深入开展社会矛盾纠纷排查化解，切实做好中小学、幼儿园安全保卫工作。健全突发事件应急管理机制，加强应急体系建设，有效预防和处置各类突发事件。搞好自然灾害预警防范，在全市所有乡镇设立气象预报显示屏。以队伍建设、办公场所建设和强化服务为重点，全面提升城市社区管理水平。加强网络管理，完善信息发布制度，规范网吧经营行为。搞好统计、体育、对台、档案、保密、民族宗教、外事侨务、防震减灾、文物保护、人民防空、地方志等工作。关心支持老龄、妇女儿童、残疾人保障等工作。

加强精神文明建设。加强社会主义核心价值体系建设，大力弘扬太行精神和纪兰精神。以更大的决心、更强的力度，完成好全国文明城市创建第二年度测评验收要求的各项任务。广泛开展文明县（市、区）、文明村（镇）、文明社区等创建活动。加强国防教育，增强全民国防观念，推进军民融合发展。加强“信用长治”建设。繁荣文化事业，创作一批反映时代风貌、群众喜闻乐见的精神产品，提升城市文化软实力。

（九）切实抓好政府自身建设。政府所作的一切，都是为了人民的幸福、劳动者的体面和社会的公平正义。我们必须紧紧围绕执政为民这一根本宗旨，切实改进政府各项工作。

加强学习，增强科学发展的坚定性。读书修身，从政立德。各级政府、各个部门的工作人员，都要认真学习履行职责必备的知识，做到真学、真懂、真信、真用。通过学习强化党性、人格、良心；通过学习增长知识、增加智慧、增强本领；通过学习不断增强工作的原则性、系统性、预见性和创造性，使一切有益的知识和文化入脑入心，沉淀在我们的血液里，融汇在从政的行为中。

转变作风，提高各项工作的实效性。牢记党和人民的重托，在其位、谋其政、负其责，努力为人民群众提供良好公共服务，为社会公平正义尽心尽力。大力弘扬“认真、团结、务实”的作风，把精力集中到抓工作、办实事、解难题、求实效上。强化大局意识，增强责任意识，提高落实能力。把今年确定的目标层层分解，明确责任主体、时限要求，强化执行、反馈、监督、考核，把任务一项一项抓实，把事情一件一件办好。

廉洁从政，强化拒腐防变的自觉性。认真贯彻落实《廉政准则》，继续深入开展煤焦领域反腐败斗争，建立健全教育、监督、惩治并重的预防腐败体系，让权力在阳光下运行。坚持勤俭办一切事业，2010年压缩政府部门经常性项目支出10%。强化对财政资金使用的监察和审计，规范政府机关公务接待，杜绝铺张浪费。坚决纠正部门和行业不正之风，严格兑现政府承诺，提高政府公信力。全体政府工作人员都要心系群众，服务人民，真正做到修身慎行、怀德自重、敦方正直、清廉自守，建设有所作为、让全市人民群众满意的政府！

各位代表，群众的期盼就是政府努力的方向。在扎实做好以上各项工作的同时，突出办好改善民生的十件实事：

一是新增集中供热面积1300万平方米。二是建成天然气入市工程。三是城镇新增就业岗位3.5万个，农村劳动力就业创业培训3.5万人。四是打通府后东街水淹地到东外环、延安北路到长治学院、延安南路到南外环的道路。五是完成南护城河和长子门桥南段黎水河治理改造工程。六是新增100辆环保型公交车。七是市区繁华路段和超市、医院等大型公共活动场所新增500个停车位。八是开工建设市老年福利服务中心；新建和改（扩）建47个社区的办公服务用房。九是在全面完成省政府农村“五个全覆盖”的同时，实施农村卫生绿化、农村文化低保、五保户集中供养、农村销售服务网络、村级组织活动场所“五个全覆盖”。十是推进新一轮“菜篮子”工程，建设20个标准化设施蔬菜生产示范园，带动发展设施蔬菜2万亩；启动“放心早餐”工程。

各位代表，人民的期望殷切而厚重，我们的使命重大而光荣。让我们在省委、省政府和市委的正确领导下，深入贯彻落实科学发展观，凝聚全市人民的智慧和力量，扎实工作，锐意进取，全面完成“十一五”规划的各项任务，为推进“四位一体”发展战略、建设富裕文明和谐新长治而努力奋斗！
